# Supplementary material for: A widespread animal communication tempo may resonate with the receiver’s brain
Source: PLoS Biol. 2026 Apr 14;24(4):e3003735. doi: 10.1371/journal.pbio.3003735 (PMC13078620; doi:10.1371/journal.pbio.3003735)
Supplement: S1 Text — Section A in S1 Text. Extended neural resonance results. Results of computational experiments on neural resonance in larger networks. Section B in S1 Text. Extended graph topology results. Comparison of network properties between networks with low-order under driving and those high-order under driving. Section C in S1 Text. Additional information on xeno-canto analysis. Provides additional information on our analysis of data from the xeno-canto database. Fig A in S1 Text. Resonance with a large network. This figure illustrates resonance curves for a large network. Fig B in S1 Text. Time-series of firefly and cricket measurements. This figure compares signals for fireflies and crickets (excerpt from the same data shown in Fig 1). Fig C in S1 Text. Example of xeno-canto data digitization. This figure demonstrates how data was extracted from samples in the xeno-canto database. Table A in S1 Text. Animal isochronous tempo data across species. This table provides the data from the Xeno-canto database. (PDF) [file pbio.3003735.s001.pdf]

S1 Text.

Supporting Information: “A widespread animal communication tempo may resonate with the receiver’s brain”

A. Extended neural resonance results

To check the robustness of our results in larger networks, we also perform simulations with $N = 100$ with varying fractions of input neurons (Fig A in S1 Text); we observe similar resonance curves both with all-to-all and random binary networks.

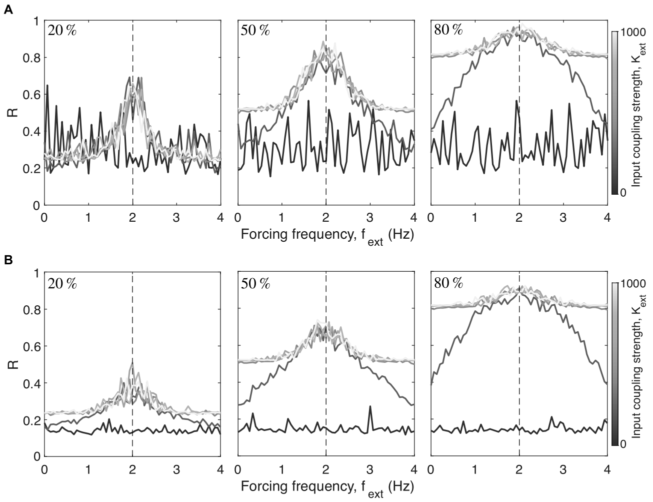

Supporting Fig. A. Resonance with a large network. Resonance curves with $N = 100$. We vary the fraction of input neurons (with 20%, 50% and 80% in separate plots from left to right). Results obtained from 1 realization (initial seed). (A) All-to-all network. (B) A random binary network, with each node assigned 60 random connections to avoid sparsity. Data and code underlying this Figure can be found in `https://doi.org/10.5281/zenodo.19069908`.

.

B. Extended graph topology results

When we attempt to ask what sets the “weaker” graph topologies apart from the others, we find that there are more reciprocal pairwise connections (i.e., if A is connected to B, B is also connected to A). In this 5-neuron example, the 10 lowest order cases have a mean of 5.38 reciprocal edges (i.e., graph reciprocity 0.54) as opposed to 5.08 in the 10 highest order cases (i.e., graph reciprocity 0.51): see Fig 4B of the main text for example graphs. The graphs also differed in degree of clustering, with a higher clustering coefficient in the lower order graphs (0.38 vs 0.32).

C. Additional information on xeno-canto analysis

Assessment of isochronous frequency for each species in the xeno-canto database was performed manually by the authors. The process consisted of (1) randomly sampling a species (Matlab’s

random number generator was used to select a page and line number from the listing of all recordings for a given category of animal), (2) looking at xeno-canto's generated spectrogram for initial assessment, (3) downloading the spectrogram if plausible signs of isochronous signaling were present, (4) loading the spectrogram into an online plot digitizer tool (we used WebPlotDigitizer), (5) clicking on periodic points visible in the spectrogram and saving the coordinates of those points (see Fig C in S1 Text for an example), (6) analyzing the resulting data (extracting IOIs and their distribution) in Matlab.

In sampling randomly, we often ran into the problem that some species or genera are heavily overrepresented in the database. After a first recording from a specific genus was included in our analysis, we rejected any subsequent randomly selected recording that came from a species in that same genus. However, following a suggestion from an anonymous reviewer, we discarded lower quality (C or D rated) recordings when higher quality (A or B rated) recordings from the same species were available. This did not appreciably change our results.

In at least one case, two recordings from the same species represented different isochronous vocalizations (e.g., an alarm call vs. a mating call), but for consistency only one was included in our analysis. In some cases it was necessary to select only a part (frequency or time range) of the spectrogram representing the signal of interest, since the isochronous portion did not persist for the whole recording or there was apparently more than one species audible for all or part of the recording. Finally, unlike in our Fig 1 C, it was hard to judge whether the data included are truly representative of the indicated species, since these data are ultimately based on public citizen-science contributions rather than scholarly peer-reviewed publications. We expect that, for these reasons and more, automatically parsing the approximately one million recordings in *xeno-canto* will prove challenging (though valuable).

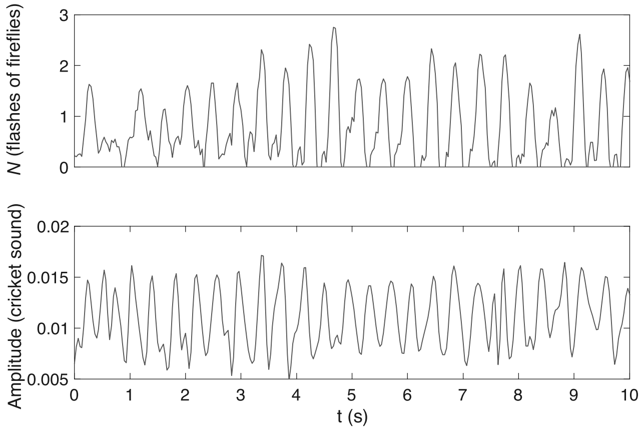

Supporting Fig. B. Time-series of firefly and cricket measurements. 10 s excerpts of the firefly summed flashing activity (top) and the sound of the cricket chirping (bottom). Data and code underlying this Figure can be found in `https://doi.org/10.5281/zenodo.19069908`.

.

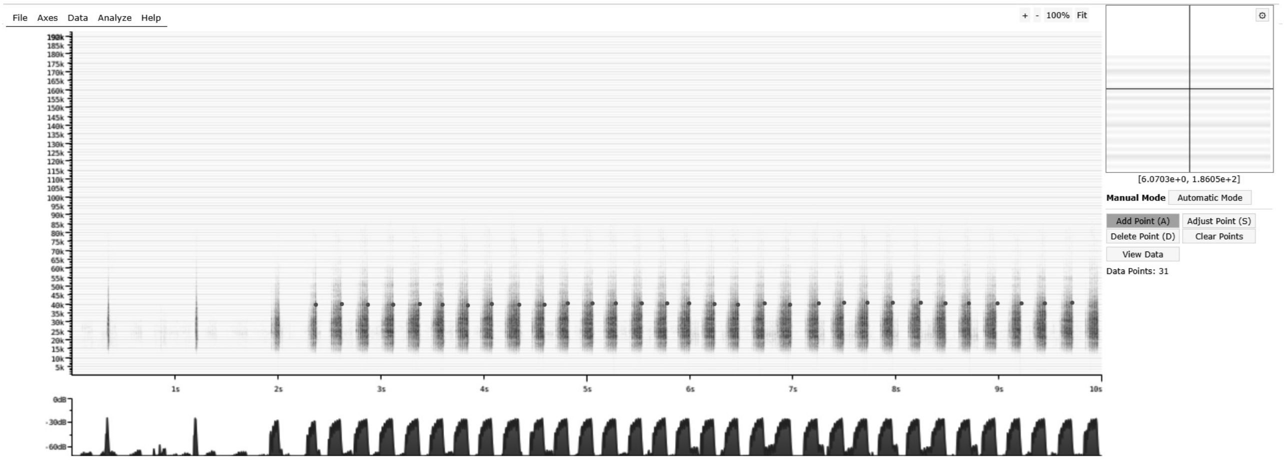

Supporting Fig. C. Example of xeno-canto data digitization. Grayscale image is directly from xeno-canto's default spectrogram. Red points show locations where user has clicked to record the start/stop of an interval. Data from Vichet's Bush-cricket *Amedegnatiana vicheti*, xeno-canto entry XC966674. Data and code underlying this Figure can be found in `https://doi.org/10.5281/zenodo.19069908`.

Supporting Table A. Animal isochronous tempo data across species. ID = Xeno-canto recording ID, N = number of intervals. * = species possibly misidentified.

ID	Common Name	Latin Name	Tempo	StdDev	StdDev%	N
Birds						
871344	Magellanic Snipe	*Gallinago magellanica*	3.69	0.21	5.6	16
36008	Chestnut-backed Thornbird	*Phacellodomus dorsalis*	3.34	0.41	12.3	21
102483	Great Spotted Woodpecker	*Dendrocopos major*	3.28	0.57	17.2	18
1063743	House Sparrow	*Passer domesticus*	2.35	0.43	18.1	22
299606	Magpie Tanager	*Cissopis leverianus*	0.56	0.04	7.3	5
623450	Short-toed Treecreeper	*Certhia brachydactyla*	4.21	0.65	15.5	8
748348	Cassin's Vireo	*Vireo cassinii*	0.65	0.13	19.3	48
988523	Fork-tailed Sunbird	*Aethopyga christinae*	1.95	0.35	17.7	37
905313	European Greenfinch	*Chloris chloris*	12.28	0.75	6.1	5
906257	Red-backed Shrike	*Lanius collurio*	1.83	0.31	16.9	9
Frogs						
882740	–	*Dendropsophus gaucheri*	1.24	0.05	4.3	10
961796	Northern Cricket Frog	*Acris crepitans*	3.82	0.16	4.3	52
891773	–	*Bufotes pewzowi*	15.50	1.15	7.4	38
919363	Black Leg Poison Dart Frog	*Phyllobates bicolor*	4.36	0.56	12.9	6
928986	–	*Chiasmocleis jacki*	5.58	0.42	7.5	11
949867	Common Toad	*Bufo bufo*	1.79	0.19	10.7	12
990053	Lemon-Yellow Tree Frog	*Hyla savignyi**	2.18	0.27	12.3	18
1019185	–	*Allobates granti*	4.14	0.37	9.0	16
1023333	Agile Frog	*Rana dalmatina*	6.00	0.41	6.9	41
928972	–	*Ameerega hahneli*	7.30	0.50	6.9	48
Grasshoppers						
756241	–	*Chorthippus vagans*	6.54	1.46	22.3	25
867517	Ambitious Meadow Bush-cricket	*Roeseliana ambitiosa*	3.90	0.58	14.8	25
873965	Tinkling Leaf-runner	*Homoeoxipha lycoides lycoides*	3.46	0.32	9.1	13
923961	–	*Platycleis intermedia*	3.33	0.25	7.6	33
820573	–	*Gomphocerus sibiricus*	4.58	0.18	3.9	38
933574	Dark Bush-cricket	*Pholidoptera griseoaptera*	0.94	0.13	13.4	61
944915	–	*Borneopsis cryptosticta*	1.99	0.42	21.0	13
966674	Vichet's Bush-cricket	*Amedegnatiana vicheti*	4.09	0.28	6.8	30
966801	Southern Saw-tailed Bush-cricket	*Barbitistes fischeri*	1.02	0.09	9.0	8
944562	Rufous Grasshopper	*Gomphocerippus rufus*	6.60	0.29	4.4	21
Land Mammals						
43407	Southern Gray Gibbon	*Hylobates muelleri*	4.66	0.57	12.3	9
638479	European Wildcat	*Felis silvestris*	0.56	0.10	18.0	6
760171	Red Fox	*Vulpes vulpes*	3.44	0.66	19.1	13
961817	Eastern Chipmunk	*Tamias striatus*	1.80	0.20	11.0	23
1009337	Toppin's Titi	*Plecturocebus toppini*	2.72	0.26	9.5	24
1016003	Bearded Capuchin	*Sapajus libidinosus*	5.80	0.76	13.0	29
1025102	Himalayan Striped Squirrel	*Tamiops mcclellandii*	2.02	0.25	12.2	9
1025105	Three-striped Ground Squirrel	*Lariscus insignis*	0.13	0.00	3.8	4
1031607	Northern Pygmy Marmoset	*Cebuella pygmaea*	1.02	0.20	20.2	11
1037186	Kenya Coast Dwarf Galago	*Paragalago cocos*	1.56	0.23	14.8	6
Bats						
821717	Seba's Short-tailed Bat	*Carollia perspicillata*	11.01	1.07	9.7	15
1016870	Eurasian Serotine	*Eptesicus serotinus*	6.94	0.91	13.0	19
843529	Common Butterfly Bat	*Glauconycteris argentata*	4.41	0.40	9.0	9
822582	Savi's Pipistrelle	*Hypsugo savii*	7.38	0.35	4.7	30
824785	Schreibers's Long-fingered Bat	*Miniopterus schreibersii*	2.72	0.59	21.7	6
822571	Daubenton's Myotis	*Myotis daubentonii*	6.91	0.56	8.1	10
934880	Common Noctule	*Nyctalus noctula*	3.37	0.65	19.2	23
1023790	Soprano Pipistrelle	*Pipistrellus pygmaeus*	2.82	0.57	20.1	21
872807	Brown Long-eared Bat	*Plecotus auritus*	4.91	0.69	14.1	20
1038063	Lesser Horseshoe Bat	*Rhinolophus hipposideros*	11.38	1.22	10.7	19
